# Integration Technology with Thin Films Co-Fabricated in Laminated Composite Structures for Defect Detection and Damage Monitoring

**DOI:** 10.3390/mi15020274

**Published:** 2024-02-15

**Authors:** Rogers K. Langat, Emmanuel De Luycker, Arthur Cantarel, Micky Rakotondrabe

**Affiliations:** 1 Laboratoire Génie de Production (LGP), University of Technology Tarbes Occitanie Pyrénées (UTTOP), University of Toulouse, 65000 Tarbes, Franceemmanuel.de-luycker@enit.fr (E.D.L.); 2 Institut Clément Ader (ICA), University of Technology of Tarbes Occitanie Pyrénées (UTTOP), University of Toulouse, 65000 Tarbes, France; arthur.cantarel@iut-tarbes.fr

**Keywords:** composites, thin films, smart composite materials, piezoelectric, sensors, structural health monitoring, polyvinylidene fluoride (PVDF)

## Abstract

Despite the well-established nature of non-destructive testing (NDT) technologies, autonomous monitoring systems are still in high demand. The solution lies in harnessing the potential of intelligent structures, particularly in industries like aeronautics. Substantial downtime occurs due to routine maintenance, leading to lost revenue when aircraft are grounded for inspection and repairs. This article explores an innovative approach using intelligent materials to enhance condition-based maintenance, ultimately cutting life-cycle costs. The study emphasizes a paradigm shift toward structural health monitoring (SHM), utilizing embedded sensors for real-time monitoring. Active thin film piezoelectric materials are proposed for their integration into composite structures. The work evaluates passive sensing through acoustic emission (AE) signals and active sensing using Lamb wave propagation, presenting amplitude-based and frequency domain approaches for damage detection. A comprehensive signal processing approach is presented, and the damage index and damage size correlation function are introduced to enable continuous monitoring due to their sensitivity to changes in material properties and defect severity. Additionally, finite element modeling and experimental validation are proposed to enhance their understanding and applicability. This research contributes to developing more efficient and cost-effective aircraft maintenance approaches through SHM, addressing the competitive demands of the aeronautic industry.

## 1. Introduction

The ongoing evolution of materials and structures, characterized by heightened functionality, reliability, and performance, has facilitated the integration of sensors, processing units, and control mechanisms at various points within structural systems or through co-fabrication processes [1]. Currently, various industrial sectors, such as aerospace, are striving for a greener future to achieve high performance with optimal energy efficiency. As a result, promising solutions such as lightweight fiber-reinforced composite materials for aerospace structures have been adopted [2,3]. Their application has resulted in reduced fuel consumption and environmental impacts [4]. There is, however, no doubt that the widespread use of these composite materials for aircraft parts, including engine fan blades, fuselage, wings, and other critical underlying components, requires the continuous monitoring of their mechanical integrity during operation, since these components are vulnerable to failure due to overloads and fabrication flaws that occur throughout the manufacturing process, in order to prevent their progression [5]. Therefore, a significant paradigm shift in the aerospace sector will result from the “structural health monitoring” (SHM) strategy, referring to a new technology that utilizes embedded sensors inside the structure to continuously and autonomously monitor the physical status of a structure with as little manual intervention as possible [6].

This strategy is realized based on the smart-material-based structural systems employing sensor technology with an integrated intelligent algorithm to assist in the collection and analysis of data on the health status of structures, thereby improving life-cycle management [7]. Moreover, the critical need for smart system deployment in the aeronautical sector necessitates the development of active composite structures. Indeed, the development of these smart structures is made possible by the high-quality features of active piezoelectric materials: their ease of integration/co-fabrication with fiber-reinforced composite structures and their capacity to be used as sensors but also actuators [8,9,10]. These materials include lead zirconate titanate (PZT), a ceramic-based piezoelectric known for its high piezoelectric charge constant, which allows for significant displacements in actuation applications. PZT also features high sensitivity, which has increased its use in sensing [11,12,13,14], Another piezoelectric material is PVDF, a polymer-based piezoelectric valued for its excellent sensitivity and greater flexibility, making it ideal for sensing applications [15,16]. The combination of smart materials, specifically piezoelectric ones, with algorithms aims to leverage the unique strengths of both tools for enhanced performance in SHM. Additionally, the technologies and processes used to produce these active materials with integrated functionalities continue to gain interest among researchers and industrial players [17,18,19,20,21]. Thanks to the existence of thin film piezoelectric materials, integration is facilitated with minimal mechanical impacts on the structure, all while boasting enhanced sensing capabilities. It is evident that composite structures with embedded sensors enable real-time monitoring, which can be used to collect useful information about the functionality and structural integrity of the composite parts during service [16,20].

Furthermore, embedded sensors allow for the monitoring of some critical and inaccessible structural parts, as well as the identification and monitoring of barely visible impact damages (BVID) in composite structures [5]. Certainly, the SHM strategy is desirable in the aeronautics industry because vital information about the health of plane structures can be established before the development of critical damages, leading to the avoidance of accidents [22,23]. SHM technology provides numerous benefits, such as increased security as a result of updates on the state of critical structures, the enhancement of condition-based maintenance (normally, the schedule maintenance technique would be applied when using the conventional non-destructive testing (NDT), which necessitates that the plane is grounded to run scheduled maintenance checks) and thus a reduction in delays in airplane maintenance, and the lowering of repair costs, which contributes to low life-cycle costs [24,25,26]. Nonetheless, commercial full-scale SHM systems are still being investigated [27], with the researchers’ main concern being the limited battery lifespan required to power these sensors, which impedes monitoring autonomy. Additionally, since these sensors are primarily intended for use inside structures, replacing their batteries could be difficult. The integration and use of active piezoelectric materials, which are essentially transducing elements, is foreseen to eliminate the need for external powering systems, thus enhancing the desired monitoring autonomy.

## 2. Related Work

The current literature reveals a significant increase in research on SHM systems for composite materials, with researchers actively investigating various methodologies, notably those utilizing integrated sensors [7,8,24,25,28,29,30,31,32,33,34,35]. For example, Paget et al. [36] conducted an illustrative study on damage detection using the Lamb wave technique by embedding a piezoceramic transducer in composites. Lamb waves, defined as elastic waves propagating in a solid plate with free boundaries [26,36], were leveraged for their exceptional sensitivity to early-stage structural deterioration, employing PZT sensors for wave transmission and reception. The study introduces methods of identifying impact damages from Lamb waves, including the amplitude-based method, which compares wave responses before and after damage, and the time-based method, which analyzes changes in Lamb wave modes over time. The study demonstrates the efficacy of these techniques in damage detection systems for composites. However, it provides limited discussion of the specific variations in local amplitude concerning the severity of damage to the structure.

Several other studies have been conducted on SHM methods with embedded piezoelectric sensors. In their comprehensive review, Philibert et al. [26] delve into Lamb-wave-based technologies for the monitoring of the structural health of composite materials in aircraft applications. The paper explores diverse methods and techniques employed in damage detection, primarily emphasizing those utilizing piezoelectric transducers. The authors introduce an intriguing damage assessment and monitoring concept, incorporating passive and active sensing approaches. In the realm of passive sensing, measurements rely on the disturbance of the structure caused by an unforeseen event, like an impact or the development of a crack. Such events typically produce elastic waves, continuously detected by sensors monitoring structural health [37]. In contrast, the active sensing approach employs an actuator to generate a controlled signal that travels through the structure. An integrated sensor captures these signals, and any deviations from the established baseline signal indicate potential growth or damage initiation [38]. However, a comprehensive study is still required to thoroughly comprehend and categorize the most effective methodologies for the analysis and detection of various types of damage based on the data collected from these seemingly promising approaches.

Masmoudi et al. [24] employed a passive sensing approach to monitor the structural health of composites, incorporating implanted piezoelectric sensors and utilizing the acoustic emission (AE) technique. This method has proven effective for real-time damage monitoring, generating transient ultrasonic waves in response to damage development within the material. In their work, they positioned the sensor in the midplane of a laminated composite structure. They subjected it to three-point bending tests with static and creep loading while continuously employing the AE technique to monitor the response. The analysis of these AE signals revealed three distinct acoustic signatures corresponding to different damage mechanisms in composites: matrix cracking, fiber–matrix debonding, and fiber breakage. This study is, however, limited to observations based on the amplitude change under static loading, not providing further features of interest in detection other than the amplitude distribution. In contrast, Chen et al. [7] applied active sensing with a fully distributed set of integrated piezoelectric sensors, utilizing the time-of-flight (ToF) technique, a method based on a wave’s propagation time measurement. Their investigation introduces the application of Fast Fourier Transforms (FFT) in analyzing signal responses to understand the impact of damage and elucidate the relationship between the response and its magnitude. However, the study confines its examination solely to the amplitude of the FFTs. It is imperative to incorporate diverse parameters to identify and classify specific damage characteristics effectively.

Therefore, in response to the growing demand for innovative solutions in the competitive aeronautic industry, this work aims to fabricate a cutting-edge smart composite structure with embedded sensors for SHM. Indeed, to compete in a highly competitive market environment, the aviation sector is actively exploring ways to reduce maintenance costs for aircraft, and full-scale SHM systems remain a subject of ongoing investigation. Eventually, this could be achieved by harnessing the potential of embedded sensors within the aircraft structure; this study, therefore, seeks to usher in a significant paradigm shift, eliminating the need for external sensors and enhancing the identification and monitoring of barely visible impact damages in composite structures, as well as significant and noticeable damages that may result from impact during operation. The paper will focus on refining SHM methods, ultimately contributing to a more efficient and cost-effective approach to aircraft maintenance in the future.

This study begins its exploration by examining the passive sensing approach, focusing on evaluating AE signals. The primary objective is identifying the optimal piezoelectric element for integration into SHM systems. The study assesses their suitability for the continuous monitoring of structural deterioration and their impact on the overall composite structure. An active sensing approach centered on Lamb wave propagation and corresponding damage diagnosis methods is introduced. An amplitude-based approach, utilizing correlation functions, is presented to gauge the damage severity. Further analysis is conducted in the frequency domain to extract multiple feature parameters for damage detection. A damage index function is formulated based on these extracted parameters. The paper also introduces a finite element modeling approach using the COMSOL Multiphysics software version 6.2, aiding in understanding the damage’s impact and the wave propagation behavior within structurally compromised systems. Finally, the experimental data are meticulously compared and validated against the results obtained from numerical analysis.

The paper is organized as follows. Section 3 presents the materials utilized in the study and the test sample manufacturing approach and introduces the conducted test methodologies and procedures. In Section 4, the results and discussion are presented with validation. Finally, Section 5 provides the conclusions obtained from the study.

## 3. Materials and Methods

### 3.1. Materials Used and the Fabrication Processes Applied

The materials considered in this study are an Epoxy E-Glass Woven prepreg (HexPly® M34/41%/300H8/G); a pre-impregnated glass fiber material with 41% of M34 resin content and a fiber volume content of 55%, developed and supplied by Hexcel Corporation (Stamford, CT, USA) (Table 1); an adhesive-based conductor in the form of copper foil tape coated with a conductive adhesive layer on one side (ADVANCE AT526), with a thickness of 35 μm; an FV301940/3 polyvinylidene fluoride (PVDF) polymer-based piezoelectric material, 52 μm in thickness, developed and supplied by Kynar, Goodfellow SARL (Lille, France) (Table 2); and a lead zirconate titanate material (PZT 5H ref. PIC 151), with a 0.2 mm thickness, also referred to as soft PZT, developed and supplied by Physik Instrumente (PI, Karlsruhe, Germany). The PZT’s properties are well described in the study of Ioan et al. [39] and presented in Table 2.

**Figure 1:**
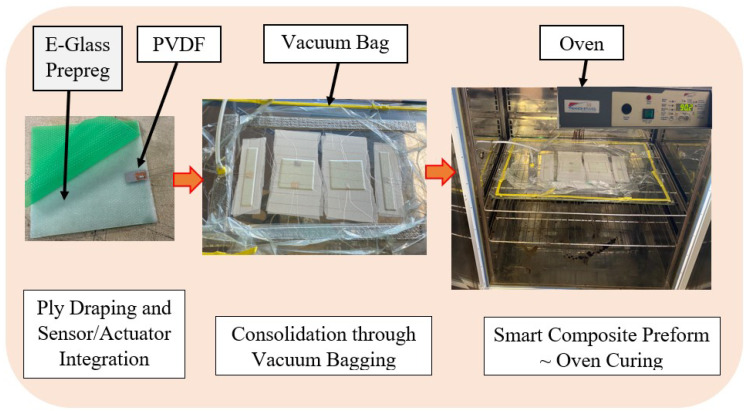
Samples’ fabrication process through consolidation molding with a curing temperature of 90 °C for 90 min.

Following a vacuum-assisted consolidation molding process, depicted in Figure 1, smart composite samples of varying sizes were fabricated by an embedding technique. The approach employed aligns with the design and fabrication processes detailed by Langat et al. [8]; nonetheless, in the present study, distinct materials and a unique wiring procedure for the active elements were utilized.

Two square plates of 100 mm × 100 mm featuring six Epoxy E-Glass prepreg plies oriented at 0° were prepared for our study. Piezoelectric material patches were embedded on both ends of the plate between the 5th and the 6th lamina. One of the square plates was composed of a 20 mm × 20 mm × 0.2 mm PZT 5H patch on one end, intended to act as a signal generator/transmitter (actuator), and on the other end was a 20 mm × 20 mm × 0.052 mm PVDF piezo film patch, anticipated to act as a sensor/receiver. The other plate featured patches of the same material, a PVDF film, measuring 20 mm × 10 mm × 0.052 mm. Additionally, two 200 mm × 30 mm smart composite beam plates were fabricated, with one featuring a 20 mm × 20 mm PZT 5H and the same size of PVDF film patch in the other sample integrated into the middle part of the beam at the same layer positions as those embedded in the square plates.

The embedded piezoelectric patches were wired utilizing adhesive-based copper conductors with tinned copper wires soldered onto them to achieve a desirable length for easy data gathering. The consolidated prefabricated smart composite plates were cured in an oven under 1 bar pressure at 90 °C for 90 min. De-molding was subsequently completed, and the resulting samples were collected for experimental tests.

### 3.2. Evaluation of the Viability of Embedded Piezo-Type Sensors for Structural Health Monitoring Using a Passive Sensing Approach and Their Impact on the Structure

**Figure 2:**
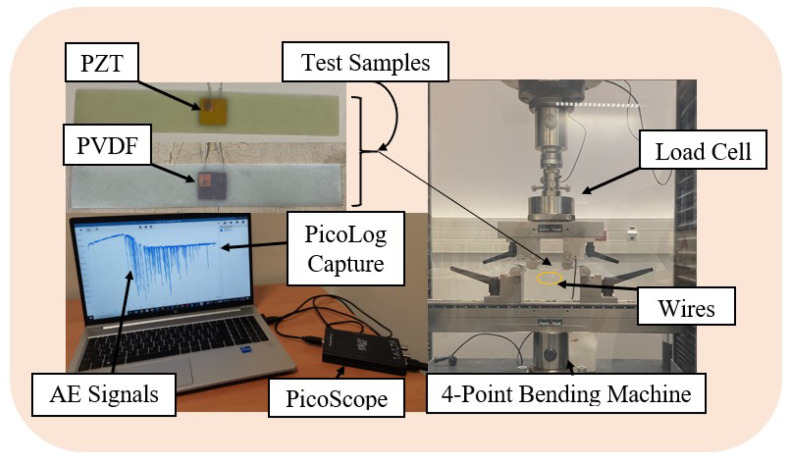
Four point bending test of the fabricated smart composite plates featuring embedded PVDF and PZT sensors to investigate passive sensing approach through acoustic emissions (AE).

The experimental setup for the analysis of the potential of the embedded PZT and PVDF patches to detect faults within fiber-reinforced composites in real time while in passive mode is shown in Figure 2. The 200 mm × 30 mm × 1.7 mm smart composite beams presented in the figure below were subjected to a 4-point bending test on the Zwick Roell Z100 material testing equipment, manufactured by ZwickRoell (Ulm, Germany) to facilitate this evaluation. The loading span was set to half of the support span, following the ASTM D6272 standard [40]. The test sample was preloaded to 1N and continually deflected with a 5 mm/min cross-head speed until it failed.

Concurrently, after reaching the preload value, PicoLog 7, a data logging software in streaming mode, was set to collect real-time data from the embedded sensor and display the response, which relied on the PicoScope 2000 series (2204A) for data acquisition. This recording provided relevant information for the SHM evaluation, including the time of material failure and the maximum flexural strength that it could withstand shortly before damage propagation. In contrast to other studies within the same framework, such as those conducted by Lampani et al. [30] and Tuloup et al. [41], which solely rely on the evolution of electrical capacitance to evaluate damage, our current work captured acoustic signals. This approach was employed to assess the suitability of both PVDF and PZT materials in detecting damage initiation. Furthermore, it was extended to verify the influence of integrating both materials into fiber-reinforced composite structures.

### 3.3. Evaluation of Actuator–Sensor Configuration in Active Sensing Approach

**Figure 3:**
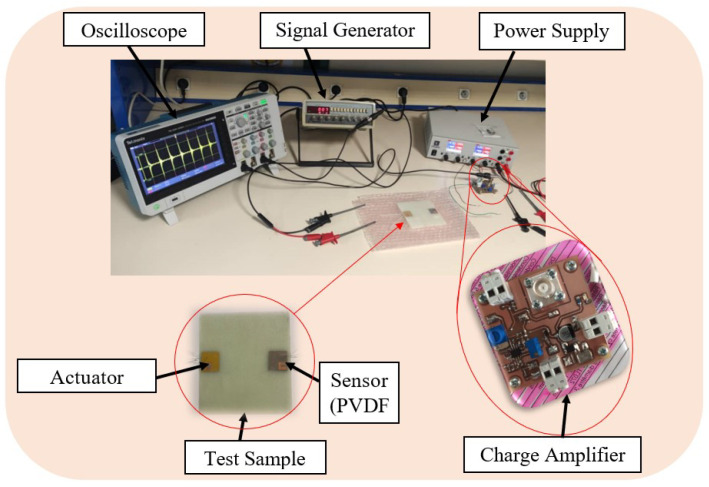
Investigation of the active sensing approach through actuator–sensor configuration (pitch-catch) based on Lamb wave propagation. A rectangular waveform with an amplitude of 31.75 V was applied to the PZT at a frequency of 6 Hz.

Figure 3 illustrates the experimental setup utilized in the active sensing approach. A 100 mm × 100 mm × 1.7 mm plate with a single pair of actuator sensors underwent testing using the pitch-catch method described in [38,42,43]. Leveraging the sensitivity of Lamb waves to structural damages like cracks, this SHM test employed an embedded PZT to generate Lamb waves. These waves were transmitted across the plate and captured by a passive PVDF sensor. The received signal served as the baseline for an undamaged structure, referred to as “Healthy” in this study. Subsequently, artificial damage in the form of a through-hole was induced, and signal responses from various damage sizes were collected. These data were then compared with the baseline of the undamaged structure, aiming to verify potential variations and assess the feasibility of the active sensing approach for SHM.

**Figure 4:**
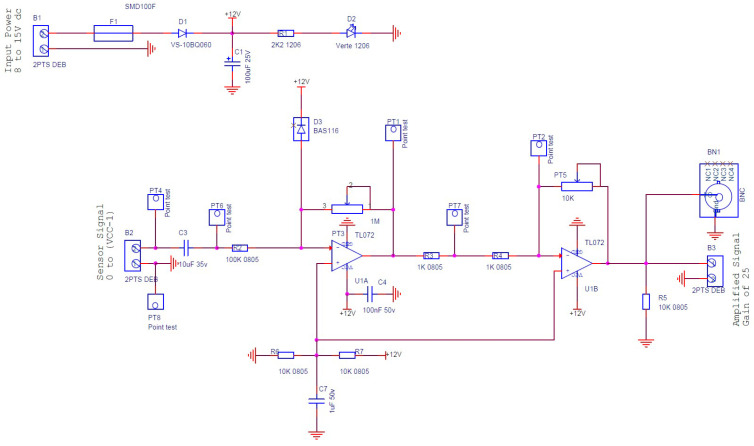
A schematic of the charge/voltage amplifier designed and manufactured to amplify the magnitude of the sensor signal.

**Figure 5:**
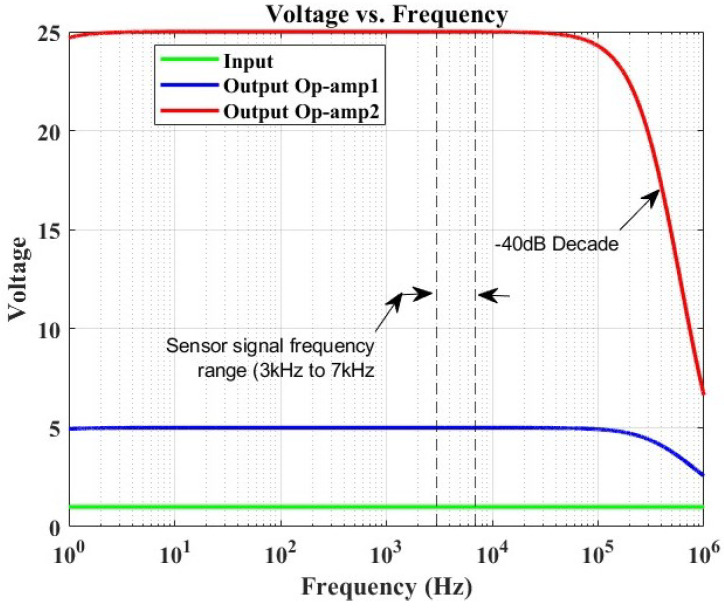
Frequency response simulation of an OrCAD-based amplifier, illustrating a voltage gain of 25 and a −3 dB frequency point at approximately 374 kHz. The sensor signal frequency range demonstrates a constant gain without any filter interference.

The equipment used in this experiment comprised a digital function generator (Topward 8112 developed by SpenceTek, Inc., Cupertino, CA, USA), a digital oscilloscope (Tektronix TBS 2104 manufactured by Tektronix, Inc., Beaverton, OR, USA), a power supply (EA-PS 2384-03B developed by EA Elektro-Automatik group, Viersen, Germany), a honeycomb optical breadboard (M-IG-32-2 manufactured by Newport Corporation, Irvine, CA, USA), and an in-house voltage amplifier, presented in Figure 4, bearing the characteristics illustrated in Figure 5.

Figure 4 illustrates the circuit of the amplifier specifically designed and manufactured for this study. The configuration consists of two cascaded operational amplifiers operating in closed-loop mode with negative feedback. This design ensures that the operational amplifier (OP-Amp) does not reach saturation and functions in the linear mode. At the input of the sensor signal, a capacitor is incorporated to eliminate any DC signal, allowing only the preservation of the AC signal response generated by the Lamb wave. Following the gain formula of the inverting amplifier (-Rf/Rin) and utilizing the given parameters, the voltage gain was determined to be 25. A simulation was conducted to determine the amplifier’s bandwidth. Figure 5 depicts the simulation results, revealing the −3 dB frequency point at 374 kHz.

The primary concern lies in evaluating the impact of the amplifier on the recorded sensor signal. The sensor signal frequencies were analyzed, indicating a frequency range of 3–7 kHz. It was observed that the amplifier did not adversely affect the signal response, as the range remained consistent throughout the frequency response evaluation of the amplifier, with no filter interference. Consequently, the amplified signal was deemed suitable for SHM investigation within the scope of this study.

### 3.4. Assessment of Finite Element Modeling (FEM) through Active Sensing

**Figure 6:**
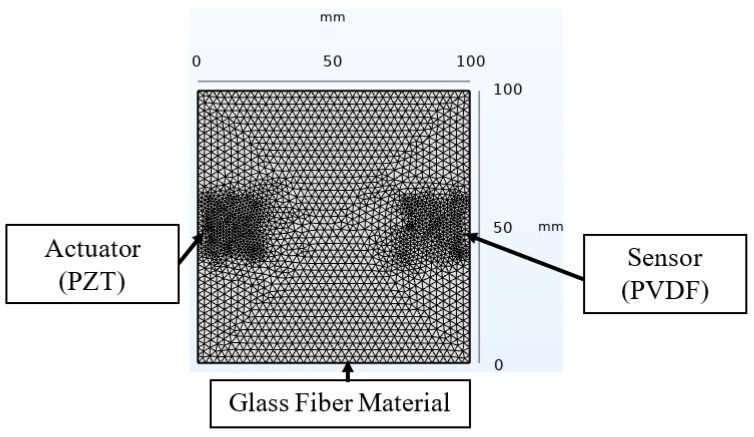
Finite element modeling of the Lamb wave propagation technique for SHM based on COMSOL Multiphysics software.

A finite element analysis based on the COMSOL Multiphysics software’s solid mechanics module was implemented to complement and validate the experimental data in the study of the active sensing SHM approach. The developed model, illustrated in Figure 6 with the associated dimensions, included defined material properties for the glass fiber (refer to Table 1). For the piezoelectric elements, materials were selected from COMSOL’s material library. The analysis assumed perfect bonding at the material interfaces in the smart composite throughout the test, ensuring that no slippage occurred between different layers [44]. In this approach, a step signal was applied to the PZT, and a domain probe captured the transient response from the PVDF sensor. The model utilized a free tetrahedral mesh with a normal density to optimize the computation time, selected after a mesh refinement approach, which showed a negligible impact on the received signal. The study, executed in the time domain, employed a fixed time step of $3.2\times 10^{-6}$ s, matching the acquisition period in the experiment. Similar damages to those induced in the experiment were investigated to analyze the variability.

## 4. Results and Discussion

This section presents the results obtained from the experimental tests conducted for both the passive and active sensing approaches, accompanied by corresponding discussions. Additionally, numerical modeling results are provided and compared with the experimental findings. Each discussion includes deductions relevant to the design and considerations for SHM strategies.

### 4.1. Passive Sensing Approach

#### 4.1.1. Mechanical Aspect

**Figure 7:**
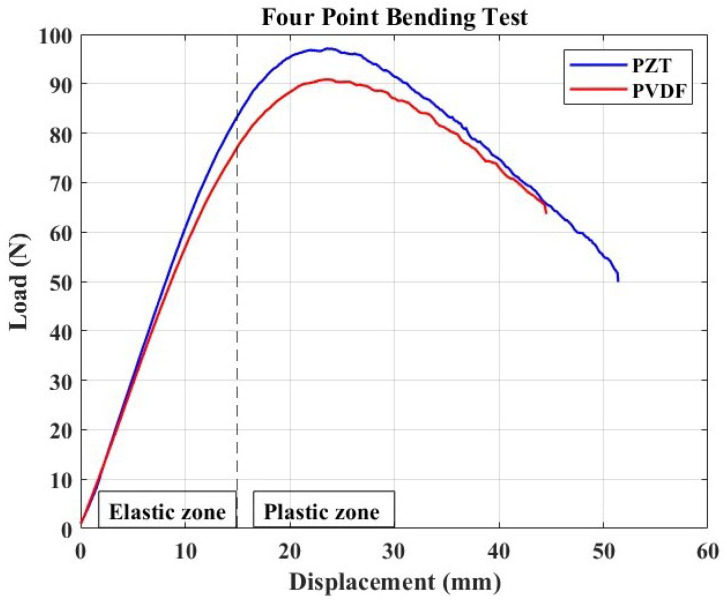
Load–displacement curve resulting from the 4-point bending test with noticeable linear (elastic) and non-linear (plastic) zone. Maximum load attained by a sample with PZT is 97.1214 N, and it is 90.8469 N for that with an embedded PVDF patch.

The load–displacement curve from the four-point bending test of the specimens with PZT and PVDF embedded is shown in Figure 7. This test was carried out solely to determine the mechanical behavior of smart composite materials when these two different types of piezoelectric materials are embedded, as well as to conclude their suitability to act as passive sensors while selecting the best that can capture the AE signal for continuous damage monitoring. This study is necessary since the literature has supported the embedding technique with the aim of protecting sensors from their surrounding environment, while creating integrated structure with smooth surfaces that could ensure, for instance, sustained aerodynamics in plane structures.

The loaded samples show two domains in the load–displacement curve: a linear elastic deformation zone followed by a plastic deformation zone with a non-linear displacement. This observation is consistent with the findings of Lampani et al.’s [30] investigation. Indeed, at the start of the test, the specimen bent without slipping on the bottom fixture. Still, as time passed, after the material’s yield strength was reached, significant slippage was observed, with the deflection curvature increasing between the two fixtures, resulting in the non-linear zone depicted in the figure. This result could also be related to the fact that, in the case of four-point bending tests of flexible materials, a zone with a rapid rupture is rarely observed since the material may fail by folding without noticeable cracks.

The sample with the PZT-based element demonstrated higher ultimate strength than its counterpart, highlighting the significant impact of the embedded piezoelectric element on the response. This phenomenon can be attributed to the implanted elements’ tendency to increase the host structure’s stiffness [8,30]. Consequently, we applied the flexural stress formula specific to a four-point bending test for our test case, where the loading point is set at half the support span. This formula is presented in Equation (1), following the ASTM D6272-2 standard test method for the flexural properties of unreinforced and reinforced plastics and electrical insulating materials by four-point bending. The maximum flexural strength determined for the PZT-based sample was 7.6174 MPa, compared to 7.1252 MPa for the PVDF-based sample. These results indicate that the PZT ceramic sample was approximately 6.9% stiffer than the one with a polymer-based piezoelectric element.

(1) $$\sigma_{f_{max}}=\frac{3F_{max}L}{4bd^{2}}$$

where $F_{max}$ is the maximum load attained, *L* is the support span, *b* is the width of the beam, and *d* is the beam thickness.

It is also critical to note the smoothness of the curve shortly after the ultimate strength point. The response becomes non-smooth at this point in the load–displacement curve, which could indicate the onset of BVID in the form of microcracks. This decision is intended to be made based on the signal acquired continually in this setup, as these damages are expected to generate elastic waves in the form of AE impacts, which may then be captured and transmitted by the passive embedded sensor.

#### 4.1.2. Sensor Performance

In the SHM approach, there is a distinct advantage to using piezoelectric materials’ electromechanical properties, which allow them to operate as transducing elements. As a result, the evaluation of the embedded piezo elements’ capacity to identify the beginning and progression of damage in composite structures was evaluated using the results of the four-point bending test, which involved continuously recording the sensor signal response in real time by the experimental setup.

**Figure 8:**
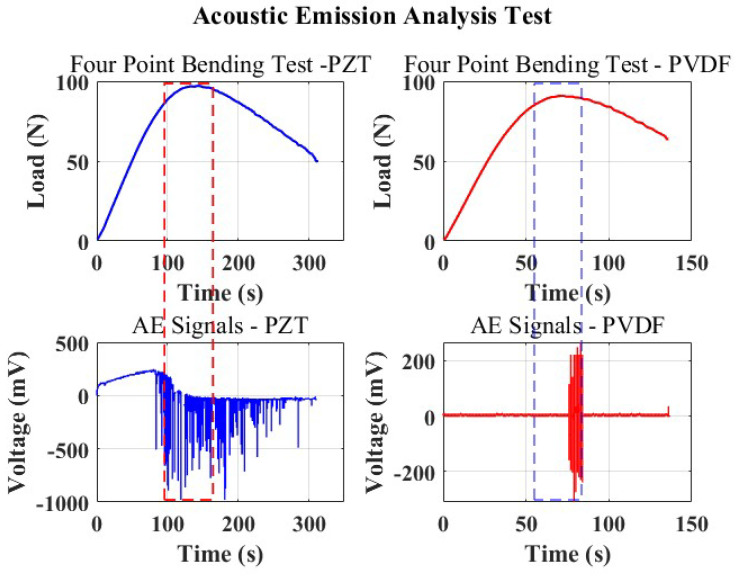
Acoustic emissions generated from the micro fractures in the loaded smart composite beam alongside the load–time curves.

Figure 8 depicts the signals of AE events recorded during the bending test, alongside the load curves plotted against time. This presentation aims to visually capture the evolution of the AE responses with reference to the mechanical behavior of the material subjected to the test.

The first curve in blue corresponds to the responses of the samples bearing PZT as a sensor. It can be seen that within the linear domain, where the beam experiences compression from the loading points with a linear displacement, the PZT experiences tension and yields a slightly linear positive response, as can be observed from the AE events captured. This could be explained by the fact that, at this point, the PZT remains compliant with the bending of the host structure. Immediately, the yield strength of the material is reached at the 90 s point, the interlaminar stresses become significant, and the displacement at this point is close to 10 mm, where the material starts to slip, beginning to create a large curvature and non-uniform displacement, leading to the bending of the PZT material as it nears the yield strength; this explains the sudden change in voltage to negative.

Realizing the nature of ceramics to be brittle, a clicking sound from the cracking PZT could be heard as the bending progressed. Despite this, the sensor remained stable, consistently capturing AE events. The commencement of damage elevated the compression forces on the PZT at the material’s maximum yield strength, resulting in peak responses. Indeed, as the bending strains weakened the beam’s mechanical performance, the sensor constantly detected AE hits. The cracking sensors suggest that the origin of microcracks within the material aligns with the embedded sensor region. This event causes a concentrated concentration of stresses, which may help to explain the unpredictable signal recorded by the PZT.

In contrast, the smart composite beam with a PVDF sensor, unlike the former, remains compliant with the loading due to its flexibility and tracks the bending of the structure while remaining sensitive to any damage. The PVDF sensor records an acoustic signal and continuously registers this signal until the load is no longer increasing as a result of the failed structure and continuous slippage of the beam. In Figure 8, it is evident that the responses from the PVDF (depicted by the red plots) exclusively capture the signals as the material surpasses its ultimate strength. This observation signifies the detection of AE signals, indicating the occurrence of actual damage to the structure.

From these observations, coupled with the previous study on the PVDF influence on the mechanical properties of the host structure [8], PVDF was concluded to have good sensing capabilities as it proved to be reliable in recording AE events only at the point of failure, and, perhaps as a result of its low electromechanical properties compared to PZT, it remained insensitive to noise. PZT, conversely, proves to be sensitive due to its high electromechanical properties; minor variations, which could be in the form of noise, are not exclusive of the received signal from its measurements.

### 4.2. Active Sensing Approach

**Figure 9:**
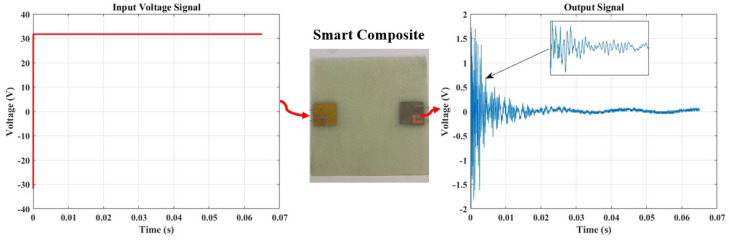
A single actuator sensor path illustrating the pitch-catch approach for damage diagnosis and monitoring, with an input signal deployed at a fixed frequency of 6 Hz.

In contrast to the passive method discussed earlier, which relies on damage-dependent signal responses for acoustic wave generation in damage monitoring and severity determination, the active sensing approach involves the continuous generation of elastic waves traveling through a material. Figure 9 illustrates our testing approach, utilizing a pitch-catch technique with a single actuator sensor configuration. In this setup, PZT generates signals by applying a step input signal, as depicted in the figure. Subsequently, a PVDF sensor receives the signals on the opposite end of the plate. The findings given in the preceding section partially influenced the selection of these components.

**Figure 10:**
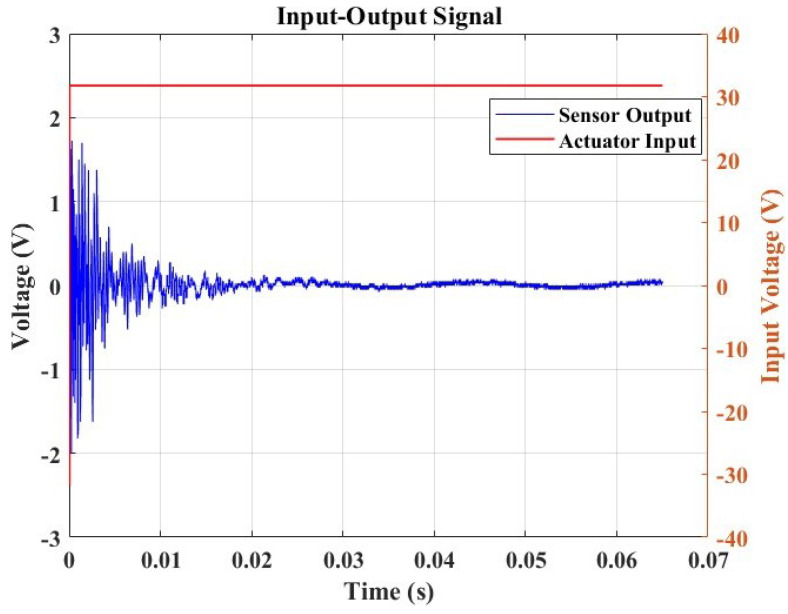
A step response from the pristine sample.

Figure 10 presents this study’s step response output signal, which will be utilized in the measurement to validate the feasibility of conducting SHM within this framework.

In this study, the Lamb wave data received from the sensor were considered for analysis in both the time domain and the frequency domain. The experiments were repeated seven times under identical conditions to ensure the reliability of the results. The goal was to understand how the signals changed and quantify the severity of the damage concerning the hole diameter’s influence. This exploration is crucial as signal variations can indicate changes in material properties, signaling the presence of damage in the structure. For the time domain study, the signal responses of varying specimen conditions were compared to the “Healthy” structure posing as the baseline. The variations in the local amplitude concerning the increase in the hole diameter were evaluated.

**Figure 11:**
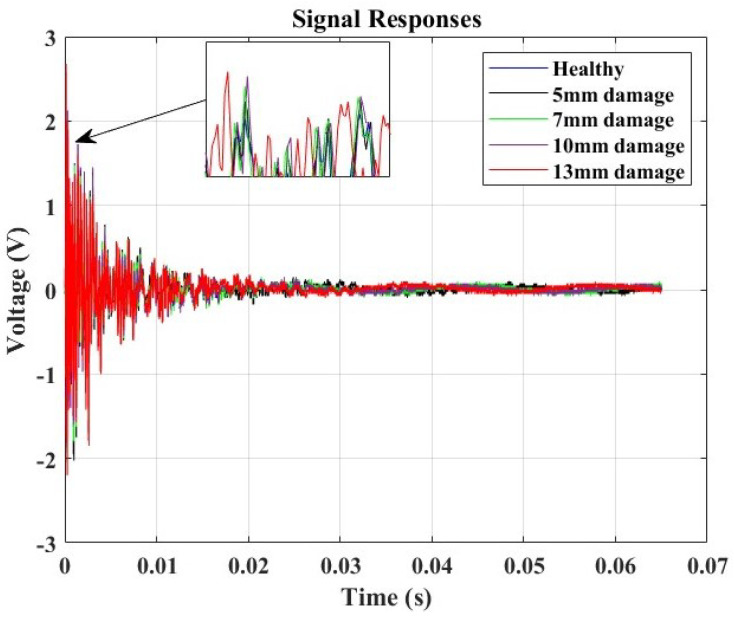
Amplitude response analysis for damage detection and correlation analysis.

Figure 11 illustrates the amplitude-based damage diagnosis approach. The collected data revealed distinct variations in peak amplitude corresponding to the different hole sizes introduced into the structure. Notably, the baseline measurement from the undamaged, healthy structure exhibited a peak amplitude of 2 V. Utilizing a relative difference metric calculated concerning this baseline, we observed a clear correlation between increasing hole sizes and higher relative differences. The peak amplitudes rose from 2.05 V for a 5 mm hole to 2.675 V for a 13 mm hole, with corresponding relative differences ranging from 0.025 to 0.3375, as illustrated in Table 3. This trend underscores the sensitivity of the monitoring system to structural changes, with larger hole sizes resulting in more pronounced deviations from the healthy baseline. The findings emphasize the efficacy of Lamb wave monitoring in detecting and quantifying structural damages, providing valuable insights for real-time structural health monitoring applications.

**Figure 12:**
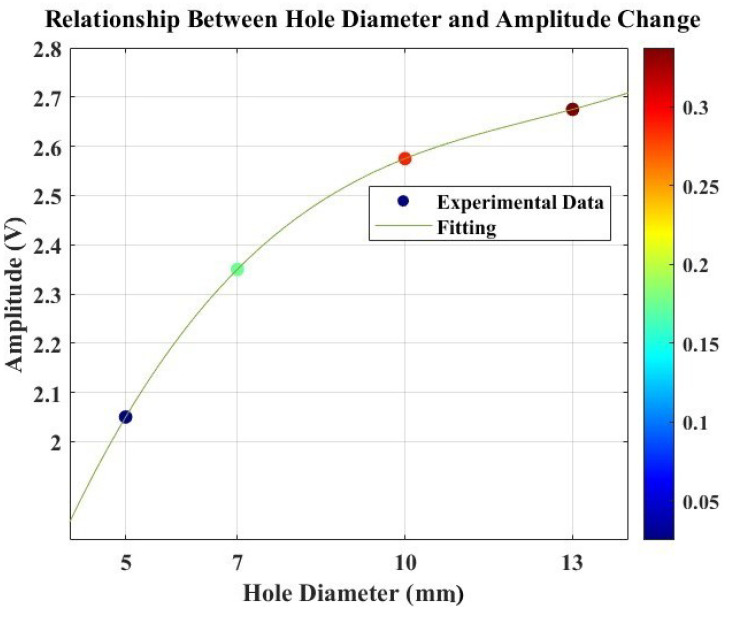
Relationship between hole diameter and amplitude responses alongside their relative differences from the baseline signal for damage diagnosis, exhibiting a non-linear relation where the amplitude of the response is influenced by the cubic power of the hole size.

Figure 12 displays the relationship between the amplitude and damage size (hole size). The plot illustrates a correlation between these variables in the structure. The observation reveals that the response amplitude follows a cubic function when a hole is introduced during the SHM test utilizing Lamb waves. This increase in amplitude can be attributed to the altered structural stiffness resulting from the introduced hole damage, facilitating easier wave propagation. To further understand this correlation, a polynomial interpolation approach was employed to fit the response ($R^{2}=1$). The resulting correlation function is presented in Equation (2), highlighting a non-linear relationship between the amplitude and hole size.

(2) $$A=0.001\lambda^{3}-0.037\lambda^{2}+0.486\lambda +0.423$$

where *A* refers to the amplitude and λ represents the hole diameter size variable.

However, this amplitude-based correlation function may not be sufficient to describe the severity of the damage in the composite structure, given the non-linear behavior of fiber-reinforced composites with alterations in the material properties; therefore, extracting more feature parameters to perform damage diagnosis and description in the composite is imperative while employing the SHM strategy. Following this determination, a further signal processing approach utilizing FFT was implemented to extract more valuable features that could best describe the presence of the damage and the influence of its size.

**Figure 13:**
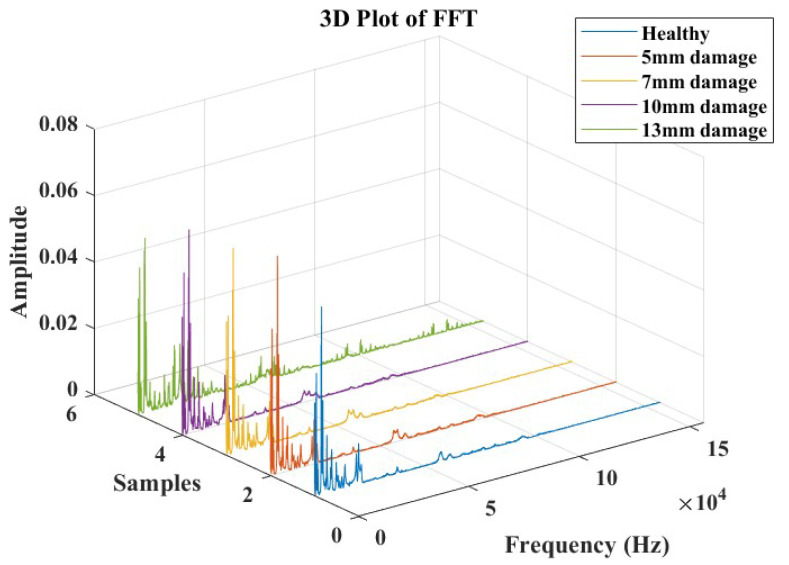
Fast Fourier Transform response analysis for damage detection and severity identification.

**Figure 14:**
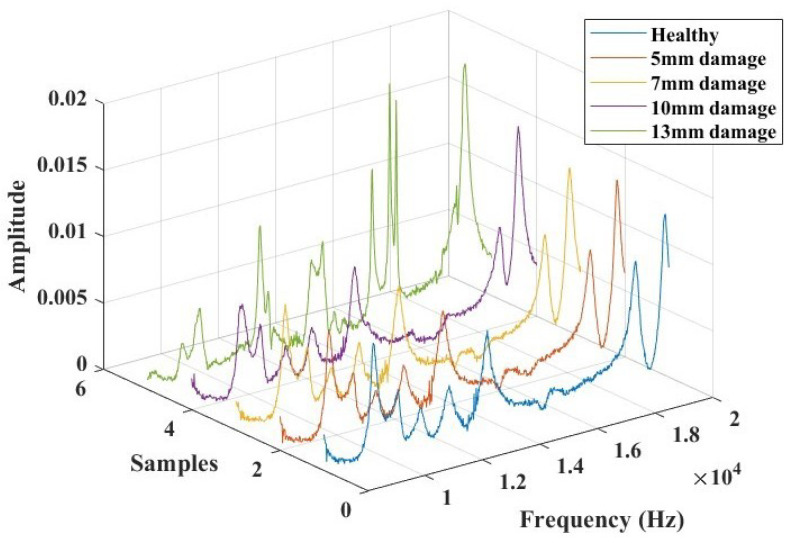
The selected frequency range (8000 Hz to 20,000 Hz) for damage identification feature extractions and analysis.

In the frequency domain, Lamb wave signals were analyzed using Fast Fourier Transforms, as illustrated in Figure 13. The time domain signals were transformed, considering a data acquisition frequency of 312 kHz. The objective of this analysis was to identify a combination of parameters that could offer an indicative correlation to the response corresponding to the presence of damage. Based on observations from these plots, a specific frequency range between 8 kHz and 20 KHz was extracted (as depicted in Figure 14), where the influence of the introduced damage on the structure appeared to be significant. This extraction was undertaken to streamline the evaluation of pertinent parameters that could offer a reliable damage index for the SHM study.

Table 4 summarizes the extracted parameters, including peak frequency values, the area under the curve, and the centroid frequency. Each of these three parameters displayed an increasing trend with the magnitude of the damage within the selected frequency range. Subsequently, the relative differences in their evolution were computed. This led to the proposal of a damage index correlation function for the diagnosis of damage in this test approach.

**Figure 15:**
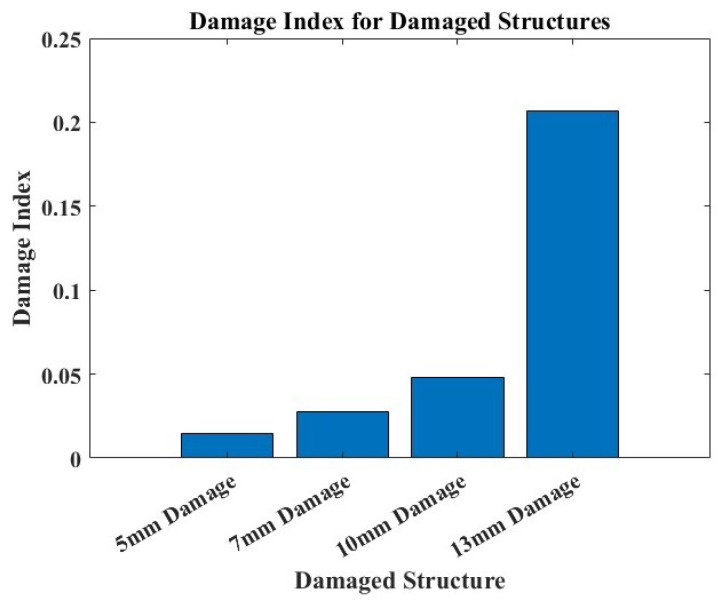
A damage index plot of the damaged structure cases.

Equation (3) illustrates the computed damage index obtained by weighting the identified relevant parameters regarding their influence on the overall damage assessment in the structure. The relevance of this established damage index lies in its ability to quantify the extent of damage or deviation from the healthy baseline. The regular computation of this damage index can form part of the proposed SHM strategy, where trends or sudden spikes in the damage indices can signal changes in structural health over time. From the observation of the data from Figure 15, it is evident that higher values of the damage index signal more significant damage, as was observed from the previous amplitude-based study correlation, where the larger relative difference values sufficed to indicate more substantial deviation from the healthy state of the composite structure.

(3) $$\mathrm{Damage}\;\mathrm{Index}=\sqrt{w_{1}\alpha^{2}+w_{2}\beta^{2}+w_{3}\gamma^{2}}$$

where $\omega_{1},\omega_{2},\omega_{3}$ are the weights for the corresponding squared relative differences, and α,β,γ represent the squared relative differences in the peak frequency, area under the curve, and centroid frequency, respectively.

A damage index plot against damaged structures in SHM is shown in Figure 15. The plot illustrates the variations in damage indices across different structures, highlighting the increasing trend with an increased damage size. The damage indices serve as a crucial indicator of structural health, facilitating the identification of deteriorating conditions that require attention. This analysis supports the implementation of condition-based maintenance strategies in aerospace structural systems.

#### Numerical Analysis

**Figure 16:**
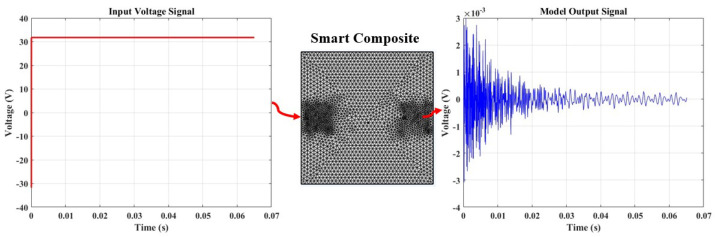
The numerical model simulation involving a step voltage input with an amplitude of 31.75 V and a fixed time step of 3.2 $\times \;10^{-6}$ s.

Figure 16 displays the outcomes of the finite element method analysis using the pitch-catch sensing approach implemented in COMSOL. The results exhibit a transient response similar in form to the one obtained from the experimental data presented in Figure 9. A similar attenuation trend is seen in the simulation’s Lamb wave signal, which converges with the experiment’s observed period. These findings complement and validate the experimental study conducted within the framework of active sensing in this work.

**Figure 17:**
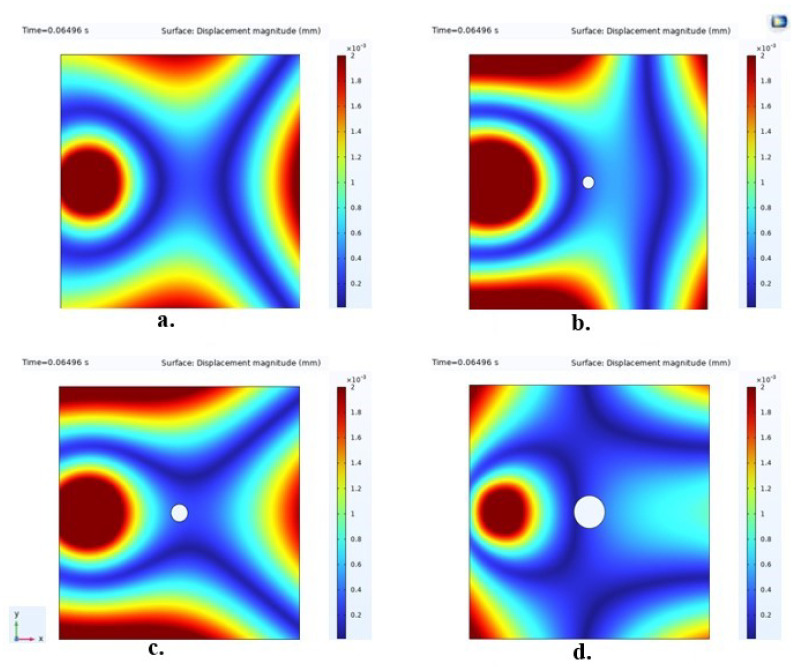
The surface plots of the analyzed sample cases displaying the displacement magnitude for visualization of the disturbance created by damage on the Lamb waves. In these representations, (**a**) illustrates the healthy structure in its intact state, (**b**) shows a structure with 5 mm hole damage, (**c**) demonstrates a structure with 7 mm hole damage, and (**d**) displays a structure with 13 mm hole damage.

The surface plots in Figure 17 illustrate displacement magnitudes with varying damage sizes. An observation reveals that, due to changes in material properties induced by damage, the Lamb wave traveling through this region undergoes scattering in multiple directions—notably, the degree of spreading increases with the severity of the impact on the structure. The signals obtained from this damage diagnosis approach are thus deemed reliable for application in implementing SHM.

**Figure 18:**
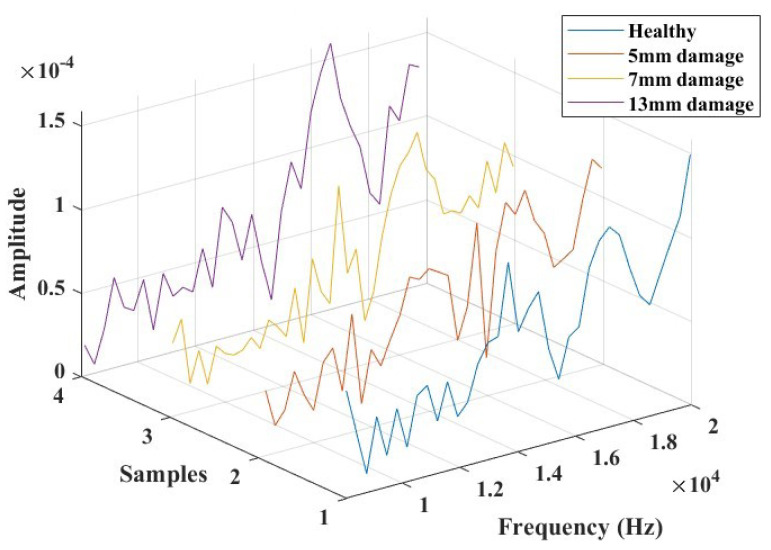
The selected frequency range for damage identification feature extractions and analysis for the simulated plate in COMSOL.

A Fast Fourier Transform of the transient response signals for the four modeled cases was performed, and a frequency range was analyzed to compute relevant parameters. Figure 18 shows the resulting plots for this frequency window. The centroid frequency feature, the center of mass of the frequency distribution, which has found interest in the study of AE signals for damage assessment [45] and structural health monitoring, was extracted as a parameter of interest. The results revealed an increasing trend in this characteristic parameter, indicating changes in structural properties.

Table 5 presents a comparison of the feature parameter between the experimental findings and FEM results. The results demonstrate strong agreement with a minimal variation of less than 2%, validating the active sensing approach for SHM.

## 5. Conclusions

This research thoroughly explored SHM methods, delving into smart composite fabrication, experimental testing, numerical analysis, and signal processing approaches. The primary objectives were to assess the suitability of thin film piezoelectric elements for SHM in fiber-reinforced composite structures and enhance the damage identification and severity classification methods.

The initial focus involved implementing an experimental study based on a passive sensing approach. The findings revealed that thin film PVDF, compared to PZT piezo materials, is well suited to the sensing of impact damages due to its low noise sensitivity and ability to capture AE signals from the onset of damage. Additionally, these sensors proved effective in analyzing the material’s load-bearing capacity. Notably, the influence of these sensors on the mechanical behavior of the host composite structure was observed, with PZT showing compromises in the flexibility of the composite structure, as indicated by the computed maximal flexural strengths.

Subsequently, a pitch-catch active sensing approach based on Lamb wave propagation was introduced, accompanied by extensive signal processing methodologies. This led to the development of a damage size correlation function and a damage index, demonstrating the high sensitivity of Lamb wave propagation to structural deterioration caused by alterations in material properties. The non-linear correlation between the damage size and severity was attributed to the complex coupling of parameters influenced by damages in composites. Combining the extracted characteristic parameters with the associated weights was considered for comprehensive damage interrogation from the signal responses.

Finally, a finite element modeling approach was employed to study the Lamb wave propagation behavior in response to damage. The numerical analysis validated the experimental data, showing good agreement with a deviation of less than 2% concerning the centroid frequency. While these investigations contribute valuable insights to ongoing studies in structural health monitoring integration technologies, it is important to note that the present study was limited to specific environmental test conditions. Future work will explore similar tests under varying environmental conditions.

## Figures and Tables

**Table 1:** Material properties of Epoxy E-Glass Woven prepreg (HexPly® M34/41%/300H8/G).

Material	Density	Area Weight	Fiber Volume Fraction	Young’s Modulus	Curing Temperature
E-Glass Woven prepreg	1800 [kg/m^3^]	300 [g/m^2^]	55 [%]	21 [GPa]	90 [°C] for 90 min

**Table 2:** Material properties of polyvinylidene fluoride (PVDF), a polymer-based piezoelectric material, extracted from Langat et al. [8], and those of lead zirconate titanate (PZT), a ceramic-based piezoelectric material.

Material	Density	Max Operating Temperature	Piezo Strain Constant	Young’s Modulus	Thickness
PVDF Film	1780 [kg/m^3^]	100 [°C]	d_31_ = 23	2–4 [GPa]	0.052 [mm]
			d_33_ = −33		
			[(10^−12^) C/N]		
PZT	7870 [kg/m^3^]	230 [°C]	d_31_ = −300	62 [GPa]	0.2 [mm]
			d_33_ = 600		
			[(10^−12^) C/N]		

**Table 3:** Relationship between hole diameter and amplitude change.

Hole Diameter [mm]	Amplitude Response [V]	Relative Difference
5	2.050	0.025
7	2.350	0.175
10	2.575	0.2875
13	2.675	0.3375

**Table 4:** Relationship between hole diameter and the extracted features in the frequency domain, showing the evolution of the damage indices.

Hole Diameter [mm]	Peak Frequency	Area under the Curve	Centroid Frequency	Relative Difference—Peak Freq.	Relative Difference—Area	Relative Difference—Centroid Freq.	Damage Indices
Baseline	19,861.7670	28.0935	29,846.5018	–	–	–	–
5	19,723.4111	28.7185	30,160.4476	−0.0070	0.0222	0.0105	0.0144
7	19,600.4280	29.3909	30,211.4998	−0.0132	0.0462	0.0122	0.0274
10	19,354.4618	30.3196	30,556.2719	−0.0255	0.0792	0.0238	0.0480
13	16,418.2409	37.3246	31,530.3745	−0.1734	0.3286	0.0564	0.2066

**Table 5:** Comparison of experimental and FEM results based on centroid frequency variation (<2% deviation).

Hole Diameter [mm]	Centroid Frequency—Simulation	Centroid Frequency—Experiment	% Difference
Intact (Healthy)	30,224.5970	29,846.5018	1.3
5	30,607.5207	30,160.4476	1.5
7	30,750.5275	30,211.4998	1.8
13	30,947.5353	31,530.3745	1.9

## Data Availability

Data are contained within the article. Additionally, the raw data are available upon reasonable request.
